# Patient-Specific Virtual Stent-Graft Deployment for Type B Aortic Dissection: A Pilot Study of the Impact of Stent-Graft Length

**DOI:** 10.3389/fphys.2021.718140

**Published:** 2021-07-26

**Authors:** Xiaoxin Kan, Tao Ma, Zhihui Dong, Xiao Yun Xu

**Affiliations:** ^1^Department of Chemical Engineering, Imperial College London, London, United Kingdom; ^2^Department of Vascular Surgery, Zhongshan Hospital, Fudan University, Shanghai, China

**Keywords:** type B aortic dissection, virtual stent-graft deployment, finite element analysis, stent-induced new entry, wall stress

## Abstract

Thoracic endovascular aortic repair (TEVAR) has been accepted as a standard treatment option for complicated type B aortic dissection. Distal stent-graft-induced new entry (SINE) is recognised as one of the main post-TEVAR complications, which can lead to fatal prognosis. Previous retrospective cohort studies suggested that short stent-graft (SG) length (<165 mm) might correlate with increased risk of distal SINE. However, the influence of SG length on changes in local biomechanical conditions before and after TEVAR is unknown. In this paper, we aim to address this issue using a virtual SG deployment simulation model developed for application in type B aortic dissection. Our model incorporates detailed SG design and hyperelastic behaviour of the aortic wall. By making use of patient-specific geometry reconstructed from pre-TEVAR computed tomography angiography (CTA) scan, our model can predict post-TEVAR SG configuration and wall stress. Virtual SG deployment simulations were performed on a patient who underwent TEVAR with a short SG (158 mm in length), mimicking the actual clinical procedure. Further simulations were carried out on the same patient geometry but with different SG lengths (183 mm and 208 mm) in order to evaluate the effect of SG length on changes in local stress in the treated aorta. Comparisons of simulation results for different SG lengths showed the location of maximum stress varied with the SG length. With the short SG (deployed in the patient), the maximum von Mises stress of 238.9 kPa was found on the intimal flap at the distal landing zone where SINE was identified at 3-month follow-up. Increasing the SG length caused the maximum von Mises stress to move away from the distal landing zone where stress values were reduced by approximately 17% with the medium-length SG and by 60% with the long SG. This pilot study demonstrates the potential of using the virtual SG deployment model as a pre-surgical planning tool to help select the most appropriate SG length for individual patients.

## Introduction

Aortic dissection is a life-threatening aortic disease. Starting with a tear in the intima of the aortic wall, it allows blood to accumulate in the media, delaminating the wall layers and resulting in the formation of a false lumen (FL). According to the Stanford classification system, aortic dissection with a primary entry tear in the descending aorta is classified as Stanford type B aortic dissection (Nienaber and Clough, 2015). One of the standard treatments to type B aortic dissection is thoracic endovascular aortic repair (TEVAR), during which a stent-graft (SG) is deployed in the true lumen (TL) in order to seal the entry tears, thereby minimising flow into the FL and promoting aortic remodelling. TEVAR procedure has shown promising outcomes in treating type B aortic dissection (Nienaber et al., 2013).

In pre-TEVAR planning, clinicians choose the SG size and landing position based on anatomical measurements and guidelines provided by the manufacturer. It is challenging to determine the optimal SG length and landing position to reduce the risk of post-TEVAR complications, such as stent-graft-induced new entry (SINE; Dong et al., 2009, 2010). SINE is rare but can lead to retrograde type A aortic dissection (RTAD) at the proximal end and/or the formation of new FL at the distal end which would require further open surgery or re-intervention (Ma et al., 2017). SG devices consist of metallic stent struts and a synthetic graft. They are designed to generate high bending stiffness than the local aorta and have a tendency to recover their original straight status after being deployed into a curved aorta. The excessive force generated by this tendency is recognised as ‘spring-back force’ and can potentially lead to SINE (Dong et al., 2010). In a recent clinical study, patients treated with shorter SGs (<165 mm) were found to have higher distal SINE incidence due to this spring-back force (Ma et al., 2017). Mechanical interactions between the SG and aorta influence the post-TEVAR biomechanical environment and can potentially determine the treatment outcome. Unfortunately, neither the biomechanical interaction nor the post-TEVAR SG configuration is available to clinicians at the pre-TEVAR planning stage.

In the past decade, promising progress has been made towards the development of finite element method (FEM)-based virtual SG deployment tools. Since the first report on FEM-based simulation of SG deployment in an anatomically realistic ascending aorta (Auricchio et al., 2013), the virtual SG deployment model has been improved to accommodate compliant wall and has shown great robustness for applications in the abdominal aorta (Perrin et al., 2015a,b, 2016). Very recently, SG deployment simulations have been applied to more complex scenarios in the aortic arch and abdominal aorta, demonstrating good accuracy in predicting SG configuration (Derycke et al., 2019, 2020).

Despite the aforementioned progress in the development and application of FEM-based methods for SG deployment in the aorta, their application in aortic dissection is lacking owing to the complex morphology that often involves a compressed TL and a mobile intimal flap. Ma et al. were among the first to report SG deployment simulations in type B aortic dissection (Ma et al., 2018; Meng et al., 2020), but their models involved several assumptions. Yuan et al. reported the first FEM-based virtual SG deployment simulation in type A aortic dissection, demonstrating its capability in predicting the immediate outcome of TEVAR (Yuan et al., 2020). However, these studies neglected the effect of pre-stress and blood pressure. Since the pre-TEVAR geometries used in these studies were reconstructed based on diastolic CTA scans, pre-stress of the aorta at diastolic pressure should be evaluated and accounted for when calculating wall deformation and stress under *in vivo* conditions.

Inspired by these recent studies, we present a pilot study of the impact of SG length using FEM-based simulation of SG deployment in a patient-specific type B aortic dissection. Our model incorporates pre-stress of the aorta, hyperelastic material property of the aorta with a separate material property for the intimal flap and design details of the SG device used in the actual TEVAR procedure. Additional simulations have been performed with two different SG lengths, and the predicted post-TEVAR stress distributions are compared to assess the impact of SG length.

## Materials and Methods

### Patient Information

**Figure 1:**
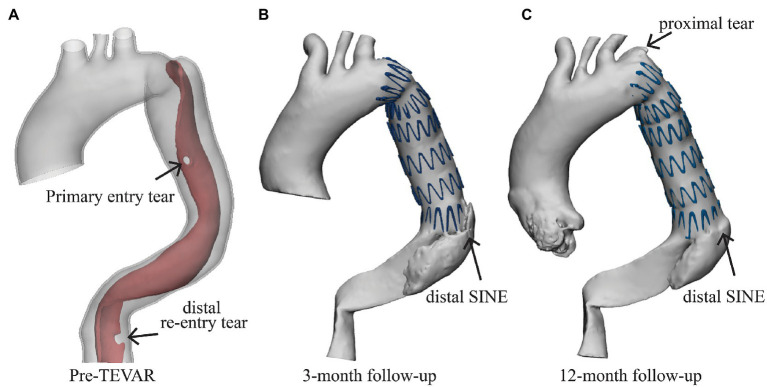
Patient information. **(A)** The patient was diagnosed with type B aortic dissection with one primary entry tear and a distal re-entry tear (marked by the black arrow), **(B)** the distal stent-induced new entry (SINE) was found at the 3-month follow-up, and (C) a newly formed proximal tear was found at the proximal landing zone at the 12-month follow-up.

A 66-year-old male patient was admitted to Zhongshan Hospital, Fudan University, Shanghai with persistent back pain. The patient underwent CTA scan using Aquilion ONE (Toshiba Medical Systems, Otawara, Japan; 1.0 mm slice thickness and 0.72 mm × 0.72 mm pixel size) and was diagnosed with type B aortic dissection (Figure 1A). Three days later, the patient underwent TEVAR intervention, during which a tapered self-expandable 42-38-158 mm Zenith 2PT SG (Cook Medical, Bloomington, Ind) was deployed into the TL and sealed the primary entry tear. Follow-up CTA scans were performed at 3-month and 12-month post-TEVAR.

A distal SINE was found at the 3-month follow-up. The new tear occurred on the intimal flap at the distal end of SG which partially pushed into the FL side (Figure 1B). On the 12-month follow-up CTA scan, the SG was found to have perforated the aortic intima and caused a minor tear at the proximal end (Figure 1C). All medical data included in this study complied with the Declaration of Helsinki and were approved by the local Ethics Committee. The patient provided written informed consent for participation.

### Aortic Dissection Modelling

**Figure 2:**
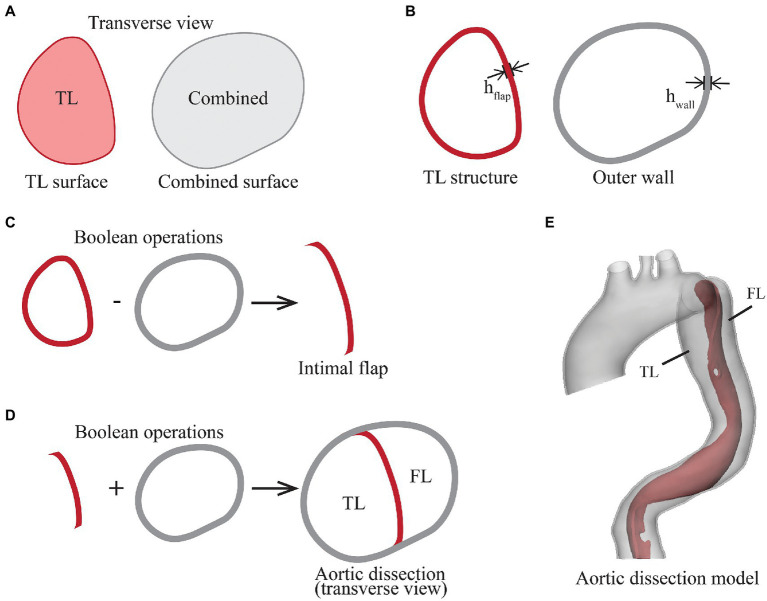
Illustration of the steps in segmentation and reconstruction of the aortic dissection geometry from pre-TEVAR CTA scan. **(A)** Transverse view of two segmentations, the true lumen (TL) surface represents the original blood flow channel, and the combined surface encloses true lumen, false lumen and intimal flap, **(B)** the TL structure and outer wall structure were created by extruding the surfaces outwardly by 1.45 mm, **(C)** the intimal flap was created by performing Boolean subtraction of outer wall from TL structure, **(D)** the TL structure and outer wall were combined, and (E) the intimal flap and outer wall geometry were smoothed and trimmed to form the aortic dissection geometry.

All CTA scans were processed using Mimics 23.0 (Materialise, Leuven). The pre-TEVAR CTA scan was segmented and reconstructed into two separate surface models: a surface model of the TL and a combined surface model representing the ‘outer surface of aortic wall’ by enclosing TL, FL and the intimal flap (Figure 2A). Following the approach described by Bäumler et al., the solid domain of aortic dissection was created by Boolean operation in Meshmixer (Autodesk, Inc.; Baumler et al., 2020).

The first step was to extrude the TL surface outwardly by a uniform thickness equivalent to the thickness of intimal flap (h_flap_), resulting in the TL structure. This was followed by extruding the combined surface outwardly by a uniformed thickness of h_wall_, resulting in the outer wall structure (Figure 2B). Boolean operation was then performed by subtracting the outer wall structure from the TL structure to obtain the intimal flap structure (Figure 2C). The lumen enveloped by the outer wall was separated into TL and FL by the intimal flap (Figure 2D). To reduce the influence of local wall thickness while focusing on analysing the impact of SG length on local aortic wall stress, both wall thickness parameters h_flap_ and h_wall_ were assumed to be constant at 1.45 mm, which corresponds to the average descending aortic wall thickness in the same gender and age group as that of the patient (Mensel et al., 2014).

The outer wall and intimal flap were further smoothed and trimmed by placing a transverse plane in the proximal ascending aorta, and cutting planes perpendicular to the local centreline in the distal segments of the supra-aortic branches and in the abdominal aorta above the aortic bifurcation (Figure 2E). The outer wall and intimal flap were meshed with tetrahedral elements [C3D4 in Abaqus^®^ (Dassault Systèmes, France)] and combined to form the aortic dissection model.

The outer wall was modelled as an isotropic, homogenous and hyperelastic material described by the second-order reduced polynomial strain energy function:

$$W=c_{10}\left({\bar{I}}_{1}-3\right)+c_{20}{\left({\bar{I}}_{1}-3\right)}^{2}$$

where $c_{10}$ and $c_{20}$ are material parameters, ${\bar{I}}_{1}$ is the first deviatoric invariant defined as

$${\bar{I}}_{1}={\bar{\lambda }}_{1}^{2}+{\bar{\lambda }}_{2}^{2}+{\bar{\lambda }}_{3}^{2}$$

where the deviatoric stretch ratios ${\bar{\lambda }}_{i}=J^{-1/3}\lambda_{i}$, i=1,2,3, with J being the total volume ratio and $\lambda_{i}$ the principal stretches. The material parameters were set to $c_{10}=150\;\mathrm{kPa}$ and $c_{20}=\;\;40\;\;\mathrm{kPa}$ based on data for healthy aorta from a previous study (Vorp et al., 2003). The intimal flap was found to behave more like a linear elastic material compared to the nonlinear elastic behaviour seen in healthy aorta (Deplano et al., 2019). Hence, the dissection flap was assumed as an isotropic, homogenous and linear elastic material with a Young’s modulus of 277 kPa and Poisson’s ratio of 0.49 (Deplano et al., 2019).

### Stent-Graft Modelling

**Figure 3:**
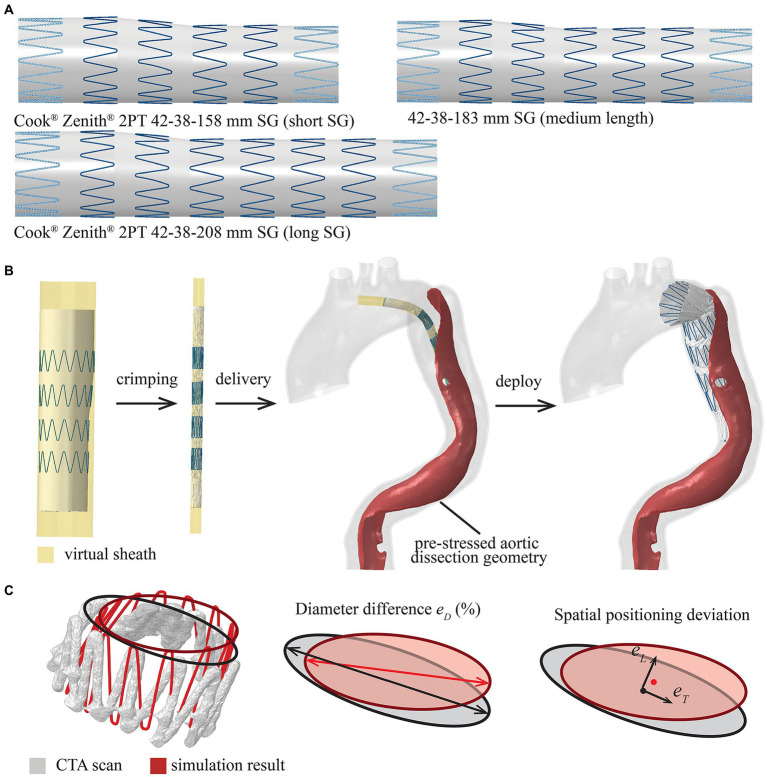
Summary of the modelled stent-graft (SG) and FEM-based simulation workflow. **(A)** Cook Zenith 2PT tapered SG with different lengths (the proximal and distal strut are shown with dash line as they were sewed inside of the synthetic graft), **(B)** the SG was crimped, delivered and deployed by controlling the virtual sheath, and (C) definitions of parameters for quantitative assessment of SG configuration, diameter difference (eD), spatial positioning deviation in longitudinal direction (eL) and transverse direction (eT).

The geometry of 42-38-158 mm Zenith TX2 PT SG (the device used to treat the patient during TEVAR), consisting of six stent struts, was created in Solidworks (Dassault Systèmes, France) by following the dimension and specification of SG from the manufacturer’s open access database (Cook Medical, 2019; Figure 3A). The next available SG length from the Zenith product library is 42-38-208 mm with eight stent struts and was created by following the design details in the same database (Figure 3A). In order to gain more insights into the impact of SG length, another medium-length SG (42-38-183 mm) with seven stent struts was artificially created by following the design features of Zenith TX2 PT product (Figure 3A). These three SG models with lengths of 158 mm (short length), 183 mm (medium length) and 208 mm (long length) were included in the virtual SG deployment simulation.

The Zenith SG was assembled by 316L stainless steel stent struts and polyethylene terephthalate (PET) fabric graft. The metallic struts were meshed into linear hexahedral elements with reduced integration (C3D8R) in Abaqus^®^ and were modelled as a linear elastic material with Young’s modulus of 210 GPa and Poisson’s ration of 0.3 (Demanget et al., 2013). The choice of using a simplified linear elastic model for the metallic struts was made because self-expandable SG opens automatically within the elastic deformation phase after the delivery sheath is removed. Based on a previous study (Demanget et al., 2013), the yield stress was never reached during SG bending. The tapered PET fabric graft was meshed into membrane elements with reduced integration (M3D4R), while the PET material was assumed to be isotropic and linear elastic with Young’s modulus of 1.84 MPa and Poisson’s ratio of 0.35 (Kleinstreuer et al., 2008). The metallic struts and synthetic graft were assembled together by using the tie constraint in Abaqus^®^. An additional cylindrical surface with a diameter of 44 mm was created outside the SG representing the delivery sheath. This virtual sheath was meshed into surface elements (SMF3D4R) for crimping, delivering and deploying the SG in the simulation.

### Numerical Simulation

Numerical simulations were performed in two steps: pre-stress of the aortic dissection model and virtual deployment of SG. Several assumptions and constraints were adopted in the simulation.

#### Kinetics and Constraints

The motion of the aortic root and ascending aorta was neglected in this study. Therefore, the nodes at the proximal ascending aorta, supra-aortic branches and distal descending aorta were fixed with zero displacement. Rayleigh damping was applied to the aorta to account for the viscoelastic tissue support on the outer arterial wall. As the descending aorta is tethered to the spine by paired intercostal arteries, this attachment was modelled by defining four pairs of fixed spots along the descending aorta, effectively preventing any excessive rigid body movement.

#### Pre-stress of the Aortic Dissection Model

The pre-TEVAR aortic dissection geometry was obtained at mid-diastole and could not be assumed as stress-free due to the intraluminal blood pressure. The calculation of pre-stress of aortic dissection was performed by modifying the method reported by Votta et al. (2017). The intraluminal blood pressure was assumed to be constant at 80 mmHg and distributed uniformly in the TL and FL. The aortic dissection model was first pressurised by increasing the internal pressure from 0 to 80 mmHg. Then, the Cauchy stress tensor calculated from the previous simulation was defined as the initial condition for the next simulation in Abaqus. The pre-stress iteration looped until the deformation of the geometry was less than 0.72 mm (the pixel resolution of pre-TEVAR CTA scan) under the 80 mmHg intraluminal pressure loading. After the iteration loop stopped, the pre-stress tensor corresponding to the mid-diastolic phase was obtained and then applied in the following virtual SG deployment simulation.

#### Virtual SG Deployment

The virtual SG deployment was performed within the pre-TEVAR aortic dissection geometry with the pre-stress tensor as the initial condition. The virtual SG deployment, including crimp, delivery and release of SG, was attained by applying nodal-specific displacement boundary conditions on the virtual sheath. In the first step, the diameter of the tubular virtual sheath was reduced from 44 mm to 8.5 mm (the diameter of introducer sheath from the manufacturer database); therefore, the SG within was compressed from its stress-free state to the crimped state (Figure 3B; Cook Medical, 2019). The contact between SG and virtual sheath was modelled by using Abaqus explicit general contact algorithm with penalty formulation. Then, the SG was bended to follow the local centreline extracted from the TL of pre-TEVAR geometry and delivered to the target landing position selected on the centreline by referring to the 3-month follow-up scan. Finally, the contact between SG and aorta was activated with the friction coefficient of 0.1 (Vad et al., 2010), and followed by the deployment of SG by expanding the virtual sheath radially to a diameter wider than the local aorta (Figure 3B). The virtual SG deployment simulation continued until mechanical equilibrium was reached. Simulations of the SG with three different lengths were performed separately with the targeting SG proximal landing position being fixed at the same location.

### Model Verification

To verify the simulation results, the SG configuration obtained from the simulation with the real SG length (short SG) was compared against the reconstructed geometry from the 3-month follow-up CTA scan by following the approach reported in a previous study (Derycke et al., 2019). The pre-TEVAR scan and 3-month follow-up scan were mapped into the same coordinate system by using the global registration algorithm in Mimics 23.0. A circle was then fitted into each stent strut end to represent the local diameter, while the centre point of the fitted circle was used to measure the spatial position of the stent strut end (Figure 3C). Differences in local diameter ($e_{D}$) between the simulation and follow-up CTA scan were calculated at each stent strut end (Figure 3C), while differences in spatial positioning of stent struts were quantified by measuring the longitudinal deviation along the aorta centreline (eL) and transvers deviation at local cross-section ($e_{T}$; Figure 3C).

## Results

The Zenith SG with three different lengths, short (158 mm), medium (183 mm) and long (208 mm), was successfully deployed into the TL in three independent simulations. After completing the virtual deployment simulations, the three SGs landed at the same proximal location, but their distal ends were at different locations. The predicted configuration of the short SG was compared against the 3-month follow-up CTA scan to verify the simulation results. Distributions of von Mises stress on the intimal flap and aortic wall were analysed and compared. Von Mises stress was chosen here as it is a measure used in material failure analysis.

### SG Configuration

**Figure 4:**
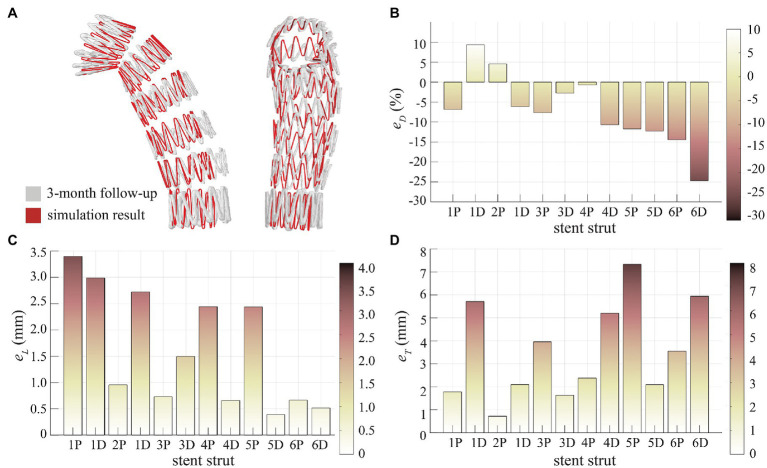
Assessment of SG configuration. **(A)** Superimposition of the SG from simulation result and 3-month follow-up scan, **(B)** diameter difference (eD) at the stent strut ends, **(C)** longitudinal positioning deviation (eL) at the stent strut ends, and (D) transverse positioning deviation (eT) at the stent strut ends.

Qualitative evaluation was firstly performed by superimposing the simulated SG configuration on top of the geometry reconstructed from 3-month post-TEVAR CTA scan (Figure 4A). The stent struts are numbered from 1 (proximal) to 6 (distal), with each strut having a proximal end (marked as P) and a distal end (marked as D). Visual inspections suggested a good agreement in the stent strut spatial position. Moreover, the opening area of the proximal SG (strut 1 to strut 3) was well reproduced by the model, but the distal segment (strut 4 to strut 6) appeared to be narrower compared to the follow-up CTA scan.

Quantitative evaluation was made by calculating $e_{D}$, $e_{L}$ and $e_{T}$ as shown in Figures 4B–D for each stent strut end. Diameter difference was −7.01 ± 9.00% for all stent strut ends (1P to 6D). Relatively small $e_{D}$ (<10%) was found for the proximal struts, but the distal strut ends (4D to 6D) had larger deviations in diameter with the model under-predicting the opening diameter by 10–25% (Figure 4B). For the spatial position of stent struts, a very good agreement was achieved in longitudinal position with $e_{L}$ being 1.62 ± 1.10 mm (Figure 4C), while the transverse spatial deviation was measured at 3.53 ± 2.09 mm (Figure 4D).

### Stress Patterns

**Figure 5:**
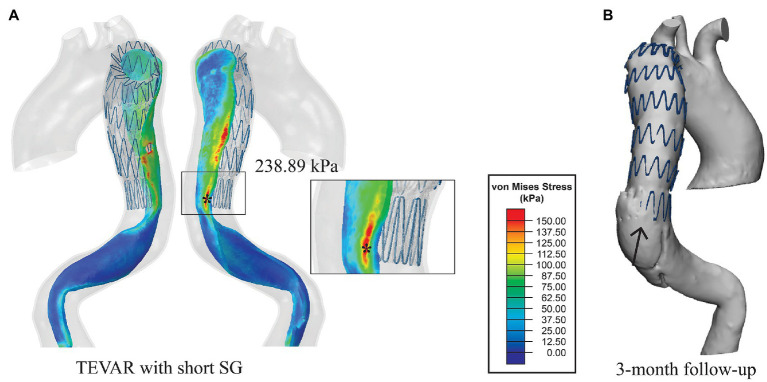
**(A)** Von Mises stress maps on the intimal flap with the short SG. The distal landing zone was zoomed in to show the local stress distribution and **(B)** the 3-month follow-up geometry from the same view angle. The distal SINE is pointed by the black arrow.

Figure 5 shows the predicted von Mises stress distributions on the intimal flap for different SG lengths. High stresses can be observed at the proximal entry tear, edge of the intimal flap and the spots where the stent strut apexes landed on. Due to local geometric discontinuity and material mismatch induced by the SG, significant difference in von Mises stress distribution can be seen in the distal area (Figure 5A).

**Figure 6:**
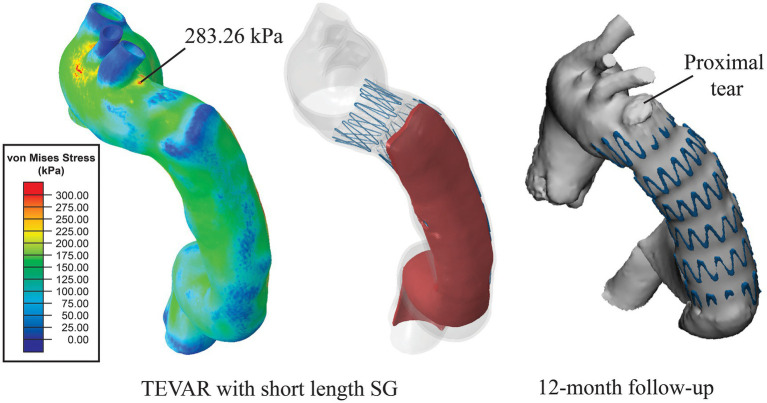
Distribution of von Mises stress on the aortic wall. High stress of 283.26 kPa was found at the location where a proximal tear was found at 12-month follow-up.

The simulation results for the short SG (mimicking the SG used in the TEVAR procedure for the patient) revealed that the maximum stress on the intimal flap was 238.89 kPa (Figure 5A), which was located at the left posterior side on the distal landing zone where the distal SINE was found on the 3-months follow-up scan (Figure 5B). Moreover, a high stress spot with a maximum value of 283.26 kPa was found on the aortic wall at the proximal landing zone where the proximal entry tear was found at 12-month follow-up (Figure 6).

**Figure 7:**
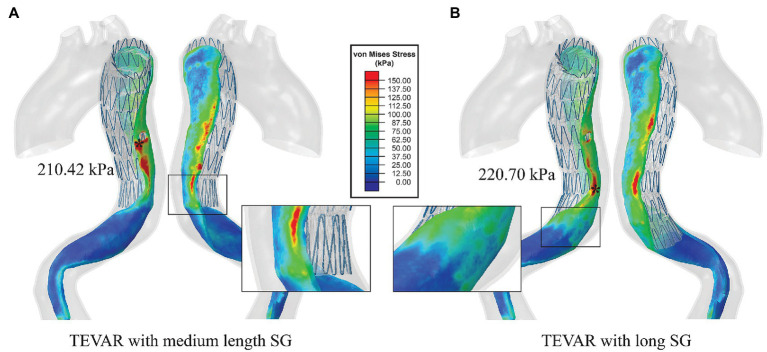
**(A)** Von Mises stress maps on the intimal flap with the medium-length SG and **(B)** von Mises stress maps on the intimal flap with the long SG. The distal landing zones of the two scenarios were zoomed in to show the local stress distribution.

Increasing the SG length resulted in changes in the stress pattern and the maximum value. With the medium-length (183 mm) SG, the maximum stress on the intimal flap was in the proximal entry tear region with a value of 210.42 kPa (Figure 7A). In the distal landing zone, elevated stress (>150 kPa) was found at the spot where the proximal end of strut 7 landed on rather than at the distal boarder of the SG. The maximum stress in the distal landing zone was reduced to 198.32 kPa (Figure 7A). With the long SG (208 mm), the maximum stress was 220.70 kPa which occurred at where the strut 6 landed on (Figure 7B), while the maximum stress in the distal landing zone was reduced to 96.69 kPa (Figure 7B).

## Discussion

Short SG has been flagged up as a risk factor for distal SINE in recent clinical studies (Li et al., 2015; Ma et al., 2017). For the clinical case presented in this study (TEVAR with 158 mm length SG), the risk assessment outcome can be contradictory based on different clinical recommendations (high risk with SG <145 mm in Li et al.’s study and <165 mm in Ma et al.’s study). Moreover, longer SG can cover more segment of the descending aorta, potentially affecting spinal cord circulation and increasing the risk for paraplegia (Nienaber et al., 2007). Further insights into the impact of SG length on post-TEVAR wall stress will be valuable for understanding the factors responsible for the formation of SINE, thereby aiding pre-surgical planning in the future.

In this pilot study, we employed a FEM-based simulation method for SG deployment in a patient-specific type B aortic dissection reconstructed from pre-TEVAR CTA scan. Previous FEM-based studies attempted to investigate the role of dissection geometry and wall stress in the occurrence of SINE (Menichini et al., 2018; Tan et al., 2021), but these studies relied on post-TEVAR CTA or magnetic resonance scans to reconstruct patient-specific geometry and assumed the SG as an additional wall layer without explicit description of the SG design. Other studies have investigated the solid-solid interaction between SG and aorta in type B aortic dissection by adopting virtual SG deployment approach, but the pre-stress conditions were ignored (Ma et al., 2018; Meng et al., 2020). By incorporating the pre-stress conditions and virtual SG deployment, our model is a step closer towards simulating the *in vivo* post-TEVAR biomechanical environment. This is important for accurate prediction of SG configuration and stress distribution.

Our simulation results were verified by comparing the deployed SG configuration with the post-TEVAR CTA scan. Ideally, imaging acquired during the TEVAR procedure or immediately after should be used for validation purpose. Unfortunately, such information was not available for this retrospective study and the earliest follow-up scan was performed at 3-month post-TEVAR. Therefore, this limitation must be factored in when evaluating the difference in SG configuration between the model prediction and CTA scan. For SG positioning, the longitudinal and transverse deviations were 1.62 ± 1.10 mm and 3.53 ± 2.09 mm (Figure 4), respectively, which are comparable to those reported by Derycke et al. with their FEM-based virtual deployment of a complex double-branched SG in a patient-specific aortic arch aneurysm (Derycke et al., 2019). However, our model had larger deviations in the SG opening diameter, especially for the distal struts with deviations up to 25%. Since the patient was found to have developed a distal SINE at 3-month follow-up, the distal end of the SG had already expanded and partially pushed into the FL side. This might explain why the predicted SG diameter at the distal end was significantly less than the 3-month follow-up configuration.

Analysis of wall stress distributions on the intimal flap and outer wall revealed that with the short SG deployed in the TEVAR procedure, the maximum von Mises stress on the intimal flap (238.89 kPa) colocalised with the region where the distal SINE occurred at 3-month follow-up (Figure 5). Furthermore, high von Mises stress was observed at the proximal landing zone on the aortic wall, coinciding with the spot where a proximal new tear was identified at 12-month follow-up (Figure 6). These focal high stress regions have shown a strong spatial correlation with the locations where the distal SINE and proximal new tear occurred. The potential link between stress concentration at the distal landing zone and distal SINE is consistent with the findings reported in patient-specific studies using a simplified finite element model (Menichini et al., 2018). By employing a virtual stenting algorithm based on simplex deformable mesh method, Chen et al. identified a correlation between large stent-induced deformation and distal SINE (Chen et al., 2018). Stress concentration at the proximal landing zone and its potential correlation with proximal new tears have also been reported in the previous studies (Ma et al., 2018; Meng et al., 2020).

In the most recent study reported by Tan et al., the effect of SG length on wall stress distribution was examined as part of a sensitivity analysis using an idealised geometric model (Tan et al., 2021). It was found that the distal SG landing position was an important factor in determining the magnitude of maximum wall stress, where landing in a straight portion of the aorta had the lowest maximum von Mises stress. However, the simplifications and assumptions made in their study meant that the complex SG design and the interactions between stent struts and intimal flap were not captured. To the best of our knowledge, no previous virtual SG deployment study has focused on the impact of SG length on aortic wall stress distribution and its correlation with SINE in type B aortic dissection.

Our simulation results showed that increasing the SG length altered the wall stress pattern, the magnitude of maximum von Mises stress and its location. With the medium-length SG, the maximum stress was found at the entry tear which was sealed by the SG. At the distal landing zone, which is vulnerable to rupture due to material mismatch, wall stress was reduced by 17% compared to the short SG. Interestingly, our simulation results for the long SG showed a further reduction of wall stress in the distal landing zone by ~60% compared to the short SG. Moreover, the maximum stress on the intimal flap was found in a region fully covered by the SG (Figure 7B).

In the clinical practice of TEVAR, SG configuration and length are usually selected based on anatomic features of the aortic dissection. With the raising awareness of post-TEVAR SINE complication, newer generation of SGs of tapered configuration and longer length are available on the market, balancing the sealing effect and SG-induced injury. Through this study, our understanding of the biomechanical behaviour of SG-induced aortic injury will help improve our ability to predict SINE risks and ultimately to assist in the development of future endograft designs and treatment planning.

## Limitation

In this study, the aortic wall was modelled as an isotropic, hyperelastic material with model parameters corresponding to healthy descending aortas. It would be desirable to use an anisotropic constitutive model with material parameters corresponding to dissected aortic tissues. The intimal flap was assumed to be linear elastic based on limited data available in the literature. Moreover, the aortic wall at the true lumen side and the false lumen side should have different material properties and wall thickness. However, direct measurement of wall thickness from CTA images was not reliable due to limited spatial resolution and the inability to distinguish between the wall and its surrounding tissues. Clearly, there is a need for comprehensive mechanical testing on dissected aortic tissues and intimal flap in the future. The influence of different material models and parameters for the aortic wall and intimal flap should also be investigated. While our virtual SG deployment model has incorporated pre-stress of the aorta, the residual stress within the tissue was not considered. Furthermore, blood flow and its interactions with the aortic wall and SG were not included in the present study, which will be addressed by combining computational fluid dynamics with the FEM-based virtual SG deployment model in the future. Finally, only one patient case was included in this pilot study, and multiple cases will be needed to obtain in-depth understanding of the impact of SG length on wall stress and its correlation with distal SINE.

## Conclusion

In this work, we applied a FEM-based virtual SG deployment model to patient-specific type B aortic dissection to investigate the impact of SG length on post-TEVAR wall stress distribution. The short SG generated high von Mises stress at both the proximal landing zone and distal landing zone where a proximal new entry tear and distal SINE were identified on follow-up scans. Using the medium-length SG reduced the wall stress in the distal landing zone by 17%, whereas a more dramatic reduction of 60% was achieved with the long SG which may potentially reduce the risk of distal SINE. This pilot study demonstrates the potential of using the virtual SG deployment model as a pre-surgical planning tool to help select the most appropriate SG length for individual patients.

## Data Availability Statement

The raw data supporting the conclusions of this article will be made available by the authors, without undue reservation.

## Ethics Statement

The studies involving human participants were reviewed and approved by Ethics Committee of Zhongshan Hospital, Fudan University, Shanghai, China. The patients/participants provided their written informed consent to participate in this study.

## Author Contributions

XK and TM: image acquisition and processing, and stent-graft modelling. XK: simulation model design, data analysis, and manuscript preparation. TM and ZD: clinical data collection. XX: supervision of the simulation work and data analysis. XX and ZD: critical revision of the manuscript. All authors contributed to the study design, revised the manuscript and approved the final submitted version.

## Conflict of Interest

The authors declare that the research was conducted in the absence of any commercial or financial relationships that could be construed as a potential conflict of interest.

## Publisher’s Note

All claims expressed in this article are solely those of the authors and do not necessarily represent those of their affiliated organizations, or those of the publisher, the editors and the reviewers. Any product that may be evaluated in this article, or claim that may be made by its manufacturer, is not guaranteed or endorsed by the publisher.
